# Time from diagnosis to surgery and prostate cancer survival: a retrospective cohort study

**DOI:** 10.1186/1471-2407-13-559

**Published:** 2013-11-27

**Authors:** Maria Theresa Redaniel, Richard M Martin, David Gillatt, Julia Wade, Mona Jeffreys

**Affiliations:** 1 School of Social and Community Medicine, University of Bristol, Canynge Hall, 39 Whatley Road, Bristol BS8 2PS, UK; 2 Bristol Urological Institute, Southmead Hospital, Bristol BS10 5NB, UK

**Keywords:** Time from diagnosis to surgery, Prostate cancer, Cancer survival, Survival inequalities, Surgery

## Abstract

**Background:**

A diagnosis of prostate cancer leads to emotional distress and anxiety, prompting calls for rapid diagnostic pathways. Nevertheless, it remains unclear what impact time between diagnosis and surgery has upon prostate cancer survival.

**Methods:**

Using national databases for England (cancer registries, Hospital Episode Statistics and Office of National Statistics), we identified 17,043 men with prostate cancer, aged 15 years and older, diagnosed in 1996–2009, and who had surgical resection with curative intent within 6 months of diagnosis. We used relative survival to investigate associations between waiting times and five- and ten-year survival.

**Results:**

Five- and ten-year relative survival estimates for the total study sample were 1.04 (95% CI: 1.04 to 1.05) and 1.08 (95% CI: 1.06-1.09), respectively. There were no notable differences in survival between patients who had surgery at 0–3 and 4–6 months after diagnosis. Relative survival was higher among the elderly (>65) and those with well and moderately differentiated tumours.

**Conclusion:**

The high relative survival in our cohort probably reflects adherence to selection criteria for surgery among men with localised prostate cancer. Among men treated with surgery within 6 months of diagnosis, we found little evidence of an association between time from diagnosis to surgery and survival.

## Background

A diagnosis of prostate cancer leads to emotional distress and anxiety [1-3], a factor which has prompted calls for rapid cancer patient pathways. However, the effects of delay between diagnosis and treatment on prostate cancer outcomes have been subject to debate. While published literature suggest that delay between diagnosis and radical prostatectomy could cause erectile dysfunction and urinary incontinence [4], its association with recurrence and survival remain unclear [5].

In attempts to decrease patient anxiety, expedite diagnosis and improve cancer survival, the UK NHS Cancer Plan (2000) and Cancer Reform Strategy (2007) were formulated [6,7]. These set maximum targets for waiting times of 14 days between fast track GP referral and first hospital appointment, and 31 days between decision to treat and start of treatment.

However, due to the comparatively lower risk of dying from prostate cancer, and the complexity of clinical management options for the disease, the effect of any waiting time standards to this site have been questioned [8]. Due to the indolent nature of most localised prostate tumours, watchful waiting and active surveillance are increasingly being offered as treatment options in the UK. Nevertheless, prostatectomy remains one of the main treatment procedures [9], and 19-35% of patients younger than 70 years of age undergo curative resection [10]. Evidence of the association of time from diagnosis to surgery on survival in prostate cancer remains unclear.

## Methods

### Data sources

The South West Public Health Observatory (SWPHO) provided us with an anonymised dataset of English cancer registry records linked to inpatient Hospital Episode Statistics (HES) and the Office of the National Statistics (ONS) mortality databases. Prostate cancer was defined as having a tumour classified in the International Classification of Diseases (ICD) version 10 as C61.

From all patients who were registered in the population-based cancer registries, patients diagnosed between January 1, 1996 and December 31, 2009, who were 15 years or older at the time of diagnosis, and who had surgical resection with curative intent within 6 months of diagnosis were included in the study. The age criterion was to allow comparability between our results and recent published survival estimates [11,12]. The 6-month cut-off was based on clinician perceptions on acceptable time from diagnosis to surgery not influenced by conditions necessitating delay (D. Gillatt, personal communications). Patients diagnosed with secondary cancers, in situ cancers, or diagnosed via death certificates only (DCO) or through autopsy were excluded.

A total of 22,152 men with prostate cancer met these criteria. From these, we excluded men with time from diagnosis to surgery of over 6 months (n = 4,171), and a further 938 patients with negative or zero post-operative survival times (follow-up). After all exclusions, we were left with 17,043 patients in the final sample.

### Study variables

The time from diagnosis to first curative surgery was defined as the time (in months) between the date of cancer diagnosis (as recorded in the registry database) and the date of the first curative resection (as recorded in HES). The date of diagnosis is defined by the cancer registries as the date of the first event or event of higher priority (if recorded within three months of the first event) among the following: histological or cytological confirmation, admission to the hospital or first consultation at the outpatient clinic because of the malignancy, or date of death [13]. In more than 99% of patients, diagnosis was confirmed through histology of the primary tumour. Time between diagnosis and surgery were categorized into 0–3 and 4–6 months.

Curative prostate cancer resections were defined as total / radical prostatectomy (M611), perineal prostatectomy (M614), open excision of prostate (M618), and prostatectomy NEC (M619), based on the Office of Population Censuses and Surveys (OPCS) Classification of Interventions and Procedures [10].

Post-operative survival was defined as the number of days between the date of the first curative resection and the date of outcome (death or censoring). Follow-up was censored at five- and ten- years, or at the end of the study period, which was December 31, 2009.

Other covariables in the study were age, region of residence, ethnicity, tumour differentiation, level of deprivation and period of cancer plan implementation. Age at cancer diagnosis was categorized as 15–54, 55–64, 65 years and above. Geographical region was defined as the patient’s region of residence at the time of diagnosis. Ethnicity was self-reported ethnicity, as recorded in the HES database [14,15]. This was categorized as White and non-White, and could not be further subdivided due to the small number of cases in ethnic groups other than White. Only ethnicity codes in 2005 to 2009 were used as these were deemed most complete (SWPHO, personal communication) [15], so ethnicity was coded as “unknown” prior to 2005. Analyses looking specifically at the effect of ethnicity on the association of time between diagnosis and surgery with survival were limited to patients diagnosed between 2005 and 2009.

Tumour differentiation refers to cell differentiation at the time of tumour biopsy and was classified as well-, moderately-, poorly- and un-differentiated. The implementation period of the UK Department of Health targets was defined as ‘prior to implementation’ (1996–2000), and ‘after implementation’ (2001–2009). Level of deprivation was derived from the income component of the 2007 Index of Multiple Deprivation (IMD) [16], and was computed for small geographical areas known as Lower Super Output Areas (LSOAs; mean population = 1500 people) [17]. Quintiles based on English IMD scores were computed, with the first quintile designated as the least deprived.

### Data analysis

The median time from diagnosis to surgery by each of the covariables were computed. Using univariable and multivariable linear regression, coefficients reflecting the additional days for each category compared to the reference category were determined for each covariable. All covariables were controlled for in the multivariable analysis. For all regression analyses, we used multiple imputation using chained equations (ICE) to account for missing data on grade and deprivation quintile [18,19]. A total of 20 complete data sets were constructed to reduce sampling variability from the imputation process [20], and the results were combined using Rubin’s rules [18,19].

Complete estimates of relative survival (where all men diagnosed between 1996 and 2009 were included, regardless of whether they had full five-year or partial follow-up) [21], were computed using the STRS command in STATA, version 12 [22]. Relative survival is a measure of survival, having accounted for underlying mortality rates. It is the ratio of the observed survival of cancer patients to the probability of survival that would have been expected if patients had experienced the same survival probability as the general population [23]. Survival probabilities were estimated at intervals of 6 months in the first year, then yearly up to 10 years. We used age-, region- and calendar year-specific UK life tables for males [24] to account for the differences in the underlying mortality and used the Ederer II method [23] to determine expected survival.

To investigate the effect of time between diagnosis and surgery on survival, the data were stratified according to categories of time to surgery (0–3 and 4–6 months) and relative survival estimates were visually inspected. To account for waiting time paradox (wherein patients offered surgery within few weeks of diagnosis could be presenting more severe manifestations of the disease), a sensitivity analyses was done using more refined time intervals (1, 2, 3–4 and 5–6 months). We limited the analysis to computing relative survival estimates because the excess mortality in the study population is negative (i.e. the mortality in our study population is lower than that of the general population) which causes model convergence problems (P. Dickman and P. Lambert, personal communication).

### Ethics/regulatory approvals

This project was approved by the Faculty of Medicine and Dentistry Committee for Ethics, University of Bristol (101153), the NHS South Central – Berkshire B Research Ethics Board (11/SC/0387) and the National Information Governance Board (NIGB, ECC 7-02(d)/2011).

### Consent

We made use of cancer registry data that were provided to us in anonymised form. The use of this data is regulated by the Confidentiality Advisory Group (CAG, formerly NIGB) of the Health Research Authority and does not require individual patient consent.

## Results

Overall, the men had a median time from diagnosis to curative surgery of 95 days (interquartile range, IQR: 70 to 125). Longer time from diagnosis to surgery were associated with increasing age, residence in the North East, Yorkshire and the Humber and the West Midlands and having well differentiated tumours (Table 1). There were no differences in time between diagnosis and surgery by ethnicity, deprivation and between the Cancer Plan implementation periods.

**Table 1:** The distribution and association of selected risk factors with diagnostic to curative surgery waiting times, prostate cancer, 1996-2009

**Variable**	**N**	**%**	**Waiting times (days)**		**Univariable analysis**		**Multivariable analysis**^**1**^	
			**Median**	**Interquartile range**	**Coefficient**^**2**^	**95% confidence interval**	**Coefficient**^**2**^	**95% confidence interval**
**Age group**								
15 - 54	1990	11.68	89	(65–119)	0.00		0.00	
55 - 64	8839	51.86	94	(70–125)	4.60	2.90 to 6.30	4.94	3.21 to 6.68
65 and above	6214	36.46	97	(70–126)	5.67	4.14 to 7.21	6.24	4.69 to 7.78
**Region of residence**								
London	1971	11.56	93	(66–124)	0.00		0.00	
North East	940	5.52	104	(80–133)	10.73	3.30 to 18.16	11.43	4.61 to 18.25
North West	1245	7.31	85	(62–114)	−6.63	−10.29 to −2.97	−6.08	−9.23 to −2.93
Yorkshire and the Humber	1914	11.23	102	(77–131)	9.07	5.38 to 12.76	9.44	6.24 to 12.63
East Midlands	1200	7.04	88	(67–115.5)	−2.79	−6.49 to 0.90	−2.78	−6.20 to 0.64
West Midlands	2012	11.81	104	(74–132)	8.88	5.17 to 12.60	9.22	6.06 to 12.38
East of England	2002	11.75	96	(73–126)	4.35	−0.75 to 9.45	6.21	2.97 to 9.45
South East	3413	20.03	91	(68–124)	0.65	−3.18 to 4.48	0.80	−2.62 to 4.23
South West	2346	13.77	91	(68–119)	−2.49	−6.19 to 1.21	−2.86	−6.19 to 0.47
**Ethnicity, major groups**^**3**^								
White	6671	68.50	90	(67–118)	0.00		0.00	
Non-White	446	4.58	94	(69–125)	3.73	−1.62 to 9.08	3.45	−1.61 to 8.51
Unknown	2621	26.92	89	(66–117)	−0.89	−3.07 to 1.30	−0.11	−2.17 to 1.94
**Tumour differentiation**								
Well differentiated	837	4.91	98	(73–130)	0.00		0.00	
Moderately differentiated	4857	28.50	98	(74–128)	−2.95	−7.02 to 1.12	−4.98	−8.53 to −1.43
Poor- & undifferentiated	1397	8.20	91	(69–117)	−7.81	−12.28 to −3.35	−9.71	−14.25 to −5.17
Unknown	9952	58.39	92	(68–124)				
**Deprivation quintile**								
1 - least deprived	4499	26.40	93	(69–125)	0.00		0.00	
2	4235	24.85	94	(69–124)	−0.37	−1.54 to 0.79	−0.26	−1.52 to 1.00
3	3477	20.40	95	(70–125)	0.33	−1.68 to 2.35	0.54	−1.34 to 2.42
4	2581	15.14	95	(70–125)	1.15	−0.83 to 3.13	1.09	−0.52 to 2.70
5 - most deprived	1737	10.19	98	(72–130)	3.67	1.63 to 5.72	2.49	0.26 to 4.72
Unknown	514	3.02	95	(65–132)				
**Cancer plan implementation period**								
Prior to implementation (1996–2000)	1701	9.98	98	(69–131)	0.00		0.00	
After implementation (2001–2009)	15342	90.02	94	(70–124)	−1.86	−5.14 to 1.42	2.91	−0.36 to 6.18

^1^adjusted for all the other variables in the table.

^2^represents the additional days waiting for each category compared to the reference category.

^3^all codes prior to 2005 were recoded as unknown; represents only data from 2005–2009.

Five- and ten-year relative survival ratio for the total study sample were 1.04 (95% CI: 1.04 to 1.05) and 1.08 (95% CI: 1.06-1.09), respectively. There were no notable differences in five- and ten-year survival between men who had surgery at 0–3 and 4–6 months (Table 2).

**Table 2:** Five- and ten-year relative survival ratio by selected risk factors and waiting time cut-offs, prostate cancer with curative surgery, 1996-2009

**Variable**	**Five-year survival**				**Ten-year survival**			
		**0-3 months waiting time**		**4-6 months waiting time**		**0-3 months waiting time**		**4-6 months waiting time**	
	**Relative survival**	**95% confidence interval**	**Relative survival**	**95% confidence interval**	**Relative survival**	**95% confidence interval**	**Relative survival**	**95% confidence interval**	
**Overall**	1.04	1.03 to 1.04	1.05	1.04 to 1.05	1.07	1.05 to 1.09	1.08	1.06 to 1.10
**Age group**								
15-54	0.99	0.98 to 1.01	1.01	0.99 to 1.01	0.99	0.95 to 1.02	1.00	0.93 to 1.03
55-64	1.02	1.02 to 1.03	1.03	1.02 to 1.04	1.03	1.00 to 1.06	1.06	1.03 to 1.08
65 and above	1.07	1.06 to 1.08	1.09	1.08 to 1.10	1.18	1.13 to 1.22	1.15	1.10 to 1.19
**Region of Residence**								
London	1.04	1.02 to 1.06	1.06	1.04 to 1.07	1.10	1.04 to 1.14	1.03	0.95 to 1.10
North East	1.03	0.99 to 1.06	1.06	1.04 to 1.07	1.13	0.99 to 1.19	1.12	1.01 to 1.17
North West	1.04	1.00 to 1.06	1.05	1.02 to 1.08	1.13	1.01 to 1.20	1.10	0.87 to 1.21
Yorkshire and the Humber	1.05	1.02 to 1.06	1.06	1.04 to 1.07	1.02	0.89 to 1.10	1.05	0.98 to 1.11
East Midlands	1.05	1.02 to 1.06	1.04	1.00 to 1.06	1.10	0.99 to 1.15	1.13	1.07 to 1.17
West Midlands	1.04	1.01 to 1.06	1.06	1.04 to 1.07	1.13	1.05 to 1.18	1.10	1.02 to 1.15
East of England	1.03	1.00 to 1.04	1.02	1.00 to 1.04	1.01	0.94 to 1.07	1.06	1.00 to 1.11
South East	1.03	1.02 to 1.04	1.05	1.03 to 1.06	1.04	0.97 to 1.09	1.10	1.06 to 1.14
South West	1.03	1.01 to 1.04	1.05	1.03 to 1.06	1.10	1.05 to 1.14	1.10	1.04 to 1.14
**Ethnicity**^**1**^								
White	1.04	1.02 to 1.05	1.06	1.04 to 1.07				
Non-White	1.08	1.04 to 1.09	1.07	1.02 to 1.08				
Unknown	1.06	1.05 to 1.07	1.07	1.05 to 1.07				
**Tumour differentiation**								
Well differentiated	1.04	1.01 to 1.06	1.05	1.03 to 1.07	1.12	1.06 to 1.16	1.12	1.07 to 1.16
Moderately differentiated	1.04	1.02 to 1.05	1.05	1.04 to 1.06	1.10	1.06 to 1.13	1.09	1.05 to 1.12
Poor- & undifferentiated	0.98	0.94 to 1.01	1.00	0.96 to 1.03	0.95	0.83 to 1.04	0.95	0.82 to 1.04
Unknown	1.04	1.03 to 1.05	1.05	1.05 to 1.06	1.06	1.02 to 1.09	1.09	1.05 to 1.12
**Deprivation quintile**								
1 – least deprived	1.04	1.03 to 1.05	1.05	1.04 to 1.06	1.07	1.00 to 1.12	1.10	1.04 to 1.14
2	1.04	1.03 to 1.05	1.05	1.04 to 1.06	1.10	1.04 to 1.14	1.12	1.07 to 1.15
3	1.03	1.01 to 1.04	1.04	1.03 to 1.05	1.05	0.97 to 1.11	1.04	0.97 to 1.09
4	1.04	1.02 to 1.05	1.05	1.04 to 1.06	1.10	1.04 to 1.15	1.09	1.02 to 1.13
5 – most deprived	1.03	1.00 to 1.05	1.06	1.04 to 1.07	1.02	0.88 to 1.10	1.12	1.06 to 1.16
Unknown	1.01	0.97 to 1.04	1.04	1.00 to 1.07	1.04	0.97 to 1.09	1.06	0.99 to 1.11
**Cancer plan implementation period**								
Prior to implementation (1996–2000)	1.03	1.00 to 1.04	1.04	1.02 to 1.05	1.05	1.02 to 1.08	1.06	1.03 to 1.09
After implementation (2001–2009)	1.04	1.03 to 1.04	1.05	1.05 to 1.06	1.08*	1.05 to 1.11	1.11*	1.09 to 1.12

*represents 9-year survival.

^1^all codes prior to 2005 were recoded as unknown; represents only data from 2005–2009.

Survival was higher with increasing age, with men 65 years and older having 8–19 percentage points increased survival compared to men aged 15–54 years. Men with well and moderately differentiated tumours had 5–15 percentage points higher survival than those with poor- and undifferentiated tumours. There was no change in five-year relative survival after the cancer plan was implemented, but a 3–9 percentage point increase in survival was observed after nine-years.

Relative survival estimates were similar even if finer time categories were used (data not shown), with the exception of patients with poor- and undifferentiated tumours. Patients with poor- and undifferentiated tumours who had surgery within one month of diagnosis had a five-year relative survival of 0.81 (95% CI: 0.56 to 0.96), indicating that the waiting time paradox applies to this group.

## Discussion

This study provides evidence that within 6 months of diagnosis, time between diagnosis and surgery does not impact on survival of men with prostate cancer. Relative survival was above 100%, indicating that these men were healthier than the general population, irrespective of age, region of residence, tumour differentiation, ethnicity, level of deprivation and whether they were diagnosed before or after the Cancer Plan implementation.

Our findings are in agreement with current literature. A recent review found no association between delay in time from diagnosis to radical prostatectomy and observed and cancer-specific survival [5]. Nevertheless, a delay of more than 9 months was reported to increase biochemical recurrence rates among men with intermediate risk disease [25], and a delay of more than 6 months was associated with disease upgrading among low-risk patients [26]. These results suggest that while delayed treatment affects cancer progression, it does not have a significant impact on survival.

Whilst most men in our study would not have received watchful waiting/active surveillance (as they all had surgery within 6 months), our data, nevertheless, reflect the findings of recent clinical trials which found little benefit of radical prostatectomy compared to watchful waiting among prostate cancer patients, at least in the medium term [27,28].

The high relative survival ratios (above 1.00) reflect the fact that men who are offered surgery are relatively fit without co-morbidities, with a realistic prospect of disease control and long life expectancy, relative to the patient’s age [9]. These criteria for surgery could likewise be the reason for better survival among elderly patients in this cohort.

In our cohort, older men (65 years and above) have higher relative survival compared to younger men (15–54 years old). This implies that older men with prostate cancer who are offered surgery are healthier and have better survival than their contemporaries in the same age group in the general population. Elderly men who are offered surgery might have less severe comorbidities, if any, and have higher life expectancy. In contrast, younger men with prostate cancer have the same level of survival compared to men of the same age group in the general population.

Our results show that factors other than waiting times may be stronger predictors of prostate cancer survival, particularly tumour differentiation. It is widely accepted that a high Gleason score (low tumour differentiation) is indicative of poorer prognosis [29]. Other factors such as stage and the presence of co-morbidities could likewise affect survival and require further research.

Our study is one of the few that have looked at the effect of time between diagnosis and surgery on prostate cancer survival, but it is not without limitations. We used routinely collected data from cancer registries and HES in England, which is known to be of high completeness and have low percentage of death certificate only cases [30]. However, it does not contain all information pertinent to patient care (PSA testing, Gleason score, stage, comorbidities and functional state), which could have explained the timeliness of treatment. We also do not have information on other forms of treatment as only Cancer registry-HES inpatient data could be provided. All our results relate to time between diagnosis and surgery not exceeding 6 months, and time interval beyond 6 months could be hazardous to the patient.

## Conclusions

Our study shows that within a period of 6 months after diagnosis, there is little evidence of an association between time from diagnosis to surgery and survival. More research is needed to fully understand the role of clinical and health care related factors in prostate cancer survival.

## Competing interests

The authors declare that they have no competing interests.

## Authors’ contributions

MTR, RM, JW and MJ conceptualized the study, MJ and RM supervised data analysis, JW and DG advised on the analysis and on the interpretation of the results, MTR analysed the data and wrote the manuscript. All authors read and agreed to the submission of the manuscript.

## Pre-publication history

The pre-publication history for this paper can be accessed here:

http://www.biomedcentral.com/1471-2407/13/559/prepub
